# Aggregation of soy protein-isoflavone complexes and gel formation induced by glucono-δ-lactone in soymilk

**DOI:** 10.1038/srep35718

**Published:** 2016-10-20

**Authors:** Sheng-Yang Hsia, Yu-Hsuan Hsiao, Wen-Tai Li, Jung-Feng Hsieh

**Affiliations:** 1 Department of Food Science, Fu Jen Catholic University, Taipei 242, Taiwan; 2 Ph.D. Program in Nutrition & Food Science, Fu Jen Catholic University, Taipei 242, Taiwan; 3 National Research Institute of Chinese Medicine, Ministry of Health and Welfare, Taipei 11221, Taiwan

## Abstract

This study investigated the glucono-δ-lactone (GDL)-induced aggregation of isoflavones and soy proteins in soymilk. High-performance liquid chromatography (HPLC) analysis indicated that isoflavones mixed with β-conglycinin (7S) and glycinin (11S) proteins formed 7S-isoflavone and 11S-isoflavone complexes in soymilk supernatant fraction (SSF). Most of the soy protein-isoflavone complexes then precipitated into the soymilk pellet fraction (SPF) following the addition of 4 mM GDL, whereupon the pH value of the soymilk dropped from 6.6 to 5.9. Sodium dodecyl sulfate polyacrylamide gel electrophoresis (SDS-PAGE) and HPLC analysis suggest that the addition of 4 mM GDL induced the aggregation of most 7S (α’, α and β subunits), 11S acidic and 11S basic proteins as well as isoflavones, including most aglycones, including daidzein, glycitein, genistein and a portion of glucosides, including daidzin, glycitin, genistin, malonyldaidzin and malonylgenistin. These results provide an important reference pertaining to the effects of GDL on the aggregation of soy protein-isoflavone complexes and could benefit future research regarding the production of tofu from soymilk.

Soybeans are often processed into a beverage referred to as soymilk. The approximate composition of soymilk includes 3.6% proteins, 2.9% carbohydrates, 2.0% fat and 0.5% ash^1^. Wagner *et al*.^2^ reported that the consumption of soymilk proteins could improve lipoprotein concentrations and reduce the progression of atherosclerosis. Soymilk proteins include two major storage proteins, β-conglycinin (7S protein) and glycinin (11S protein), which account for approximately 70% of the total protein content^3^. More specifically, the 7S protein accounts for approximately 30% of the total protein in soy and comprises the three following subunits: 7S α’ (57–72 kDa), 7S α (57–68 kDa) and 7S β (45–52 kDa). The subunits associate via hydrophobic and hydrogen bonding without any disulfide bonding^4^. The hexameric 11S protein comprises approximately 40% of the protein in soy. The dissociation of 11S depends on pH and ionic strength as well as a low content of α-helix, relatively high β-conformation, and a high contents of arginine and glutamic and aspartic acids^5^,^6^,^7^. 11S consists of acidic and basic polypeptides^8^. Each constituent subunit of 11S is composed of an acidic (A) polypeptide with a molecular weight of approximately 33 kDa and a basic (B) polypeptide with a molecular weight of approximately 20 kDa, which are linked together by an interchain disulfide bond. The isoelectric points of 7S α′, 7S α, 7S β, 11S A1a, 11S A1b, 11S B1a, 11S B1b, 11S A2, 11S A3 and 11S A4 subunits are at pH values of 5.23, 5.07, 5.88, 5.78, 5.28, 5.46, 5.73, 5.46, 5.60 and 5.29, respectively^9^.

Soymilk can be transformed into gelatinous soybean curd, which is referred to as tofu^10^. Traditionally, glucono-δ-lactone (GDL) is used as a coagulant in the industrial preparation of tofu. GDL is a carbohydrate that contains a lactone group. It hydrolyzes gradually in water to form gluconic acid, causing a reduction in pH^11^. Altering the pH value in protein solution to reach the isoelectric point leads to the formation of a protein gel through electrostatic repulsion associated with the aggregation of proteins^12^.

Tofu is also an excellent source of isoflavones, which have received considerable attention due to their antioxidant properties, estrogenic activity and cancer preventing properties^13^,^14^. A total of twelve isoflavones have been found and can be classified into four groups: (1) aglycones (daidzein, genistein and glycitein); (2) glycoside isoflavones (daidzein, genistin and glycitin); (3) glucosides that can be esterified at the 6”-*O*-position of the glucose ring with acetyl or (4) malonyl groups, forming another six compounds, known as acetyldaidzin, acetylgenistin, acetylglycitin, malonyldaidzin, malonylgenistin and malonylglycitin^15^. One previous study reported that calcium chloride induced the coagulation of β-conglycinin, glycinin and isoflavones in soymilk^16^. The addition of 5 mM calcium chloride induced the coagulation of most of the 7S (α’, α and β), 11S acidic (A1a, A1b, A2, A3 and A4) and 11S basic (B1a) proteins was well as isoflavones, including daidzein and genistein. The results clearly demonstrate that isoflavones (including daidzein and genistein) bind to 7S and 11S to form 7S-isoflavone complexes and 11S-isoflavone complexes in soymilk. These soy protein-isoflavone complexes then coagulate into SPF following the addition of calcium chloride. The presence of soy protein-isoflavone complexes suggests that β-conglycinin, glycinin and isoflavones are coprecipitated into tofu during the tofu-making process.

Although the soymilk proteins are aggregated by GDL, it is unclear how the isoflavones precipitate into the tofu during the production process. To investigate the effect of GDL on the aggregation of 7S-isoflavone and 11S-isoflavone complexes, sodium dodecyl sulfate polyacrylamide gel electrophoresis (SDS-PAGE) and high-performance liquid chromatography (HPLC) were conducted. Therefore, the objective of this study was to investigate the GDL-induced aggregation of 7S, 11S and isoflavones in soymilk.

## Results and Discussion

### Effects of GDL on the aggregation of soymilk proteins in soymilk

**Figure 1:**
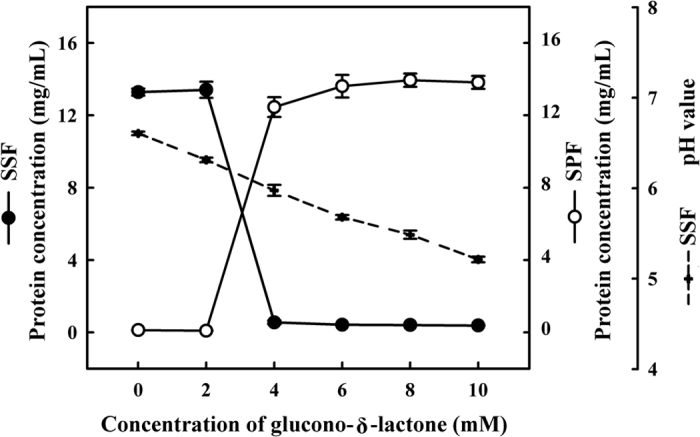
Changes in total protein content and pH of soymilk samples treated with different concentrations of GDL (0, 2, 4, 6, 8 or 10 mM). SSF: soymilk supernatant fraction; SPF: soymilk pellet fraction. Vertical bars represent standard deviations.

Soymilk samples were incubated with various quantities of GDL (0, 2, 4, 6, 8 or 10 mM) at 85 °C for 45 min. As shown in Fig. 1, the quantities of protein concentration in the SSF and SPF without GDL were 13.28 ± 0.19 and 0.12 ± 0.01 mg/mL, respectively. This suggests that the aggregation of soy proteins does not occur in soymilk samples in the absence of GDL. However, an increase in GDL concentration led to a decrease in the quantity of proteins in the SSF and an increase in the SPF. Approximately 90.1% of the soymilk proteins were aggregated into SPF following the addition of 4 mM GDL, and the total quantity of proteins increased from 0.12 ± 0.01 to 12.45 ± 0.54 mg/mL. Specifically, treatment with GDL had the following effects on pH values in the SSF samples: 0 mM (pH 6.6), 2 mM (pH 6.3), 4 mM (pH 5.9), 6 mM (pH 5.6), 8 mM (pH 5.4), or 10 mM (pH 5.2). These results indicated a significant increase in the quantity of soy proteins in the SPF following the addition of 4 mM GDL (*P* < 0.05), which resulted in a pH value of 5.9. Kohyama *et al*.^17^ reported that GDL is an acid precursor capable of cleaving gluconic acid within the solution and then dissociating gluconic acid molecules in the generation of protons, which in turn lowers the pH of the soymilk. As the pH approaches the isoelectric point of soymilk proteins, the random aggregation of neutralized proteins results in the formation of a stable gel through hydrophobic interactions and the formation of hydrogen bonds.

As previously mentioned, the isoelectric points of 7S α′, 7S α, 7S β, 11S A1a, 11S A1b, 11S B1a, 11S B1b, 11S A2, 11S A3 and 11S A4 subunits are at pH values of 5.23, 5.07, 5.88, 5.78, 5.28, 5.46, 5.73, 5.46, 5.60 and 5.29, respectively. This is an indication that this pH value approaches the isoelectric point of soymilk proteins, leading to protein aggregation. Ringgenberg *et al*.^18^ reported that as the pH of soymilk protein suspensions decreases to values close to 6, it has been observed that the basic subunits of 11S and the β subunit of 7S are the first to destabilize. As the system approaches a net neutral charge, the acidic subunits of 11S and the α and α’ subunits of 7S also begin to participate in the network formation of acid-induced gels. The driving forces behind the acid gelation of soymilk proteins are non-covalent in nature, and include salt bridging and short range interactions, such as hydrogen bonding and Van der Waals forces^19^. As previously stated, the addition of GDL to soymilk results in a reduction in soymilk pH. Therefore, our results demonstrate that the addition of 4 mM GDL (pH 5.9) indirectly causes soymilk proteins to aggregate via the direct acidification of the soymilk.

### Analysis of the effects of GDL on 7S and 11S proteins using SDS-PAGE

**Figure 2:**
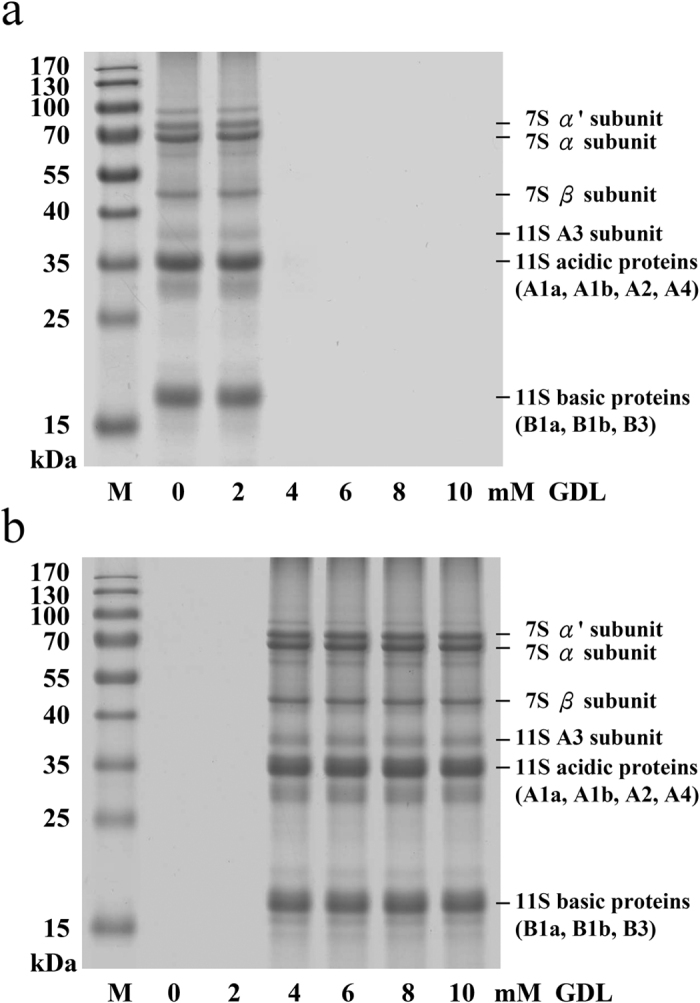
Changes in the SDS-PAGE profiles of soymilk samples treated with various concentrations of GDL (0, 2, 4, 6, 8, or 10 mM) at 85 °C for 45 min: (**a**) Soymilk supernatant fraction (SSF); (**b**) Soymilk pellet fraction (SPF); M: protein marker. Identical volumes of each sample (4 μL) were withdrawn and loaded into separate wells. Following electrophoresis, the gels were stained using Coomassie Brilliant Blue R-250. Each sample was performed in triplicate.

SSF and SPF samples with different concentrations of GDL (0, 2, 4, 6, 8 or 10 mM) were analyzed by SDS-PAGE. As previously mentioned, 7S and 11S are the two major soymilk proteins. The 7S globulin conglycinin is a trimeric glycoprotein consisting of three types of subunits: α’, α and β. Glycinin is an 11S globulin occurring as a hexamer consisting of six subunits^20^. On the basis of the SDS-PAGE results (Fig. 2), the fraction corresponding to a molecular weight of 37 kDa indicates that the protein band represents an acidic protein (11S A3). The 35-kDa fraction contained 11S (acidic) proteins, including 11S A1a, 11S A1b, 11S A2 and 11S A4. Furthermore, the 17-kDa fraction contained 11S B1a and other 11S (basic) proteins. SDS-PAGE separated the 7S α′ subunit, 7S α subunit, 7S β subunit, 11S A3 subunit, 11S acidic protein, and the 11S basic protein subunits in soymilk. The 7S α′, 7S α, 7S β and 11S A3 proteins, the 11S acidic protein, and the 11S basic protein subunits nearly disappeared from the SSF (Fig. 2a) but appeared in the SPF following the addition of 4 mM GDL (Fig. 2b). Our findings revealed a drop in pH to 5.9 following the addition of GDL, and the soymilk protein particles neutralized by acidification reached the isoelectric point, which led to the formation of a gel network. These findings are in agreement with Ringgenberg *et al*.^18^, who induced the acidification of soymilk using GDL, wherein gel aggregation occurred at approximately pH 5.8. Here the value is 2–4 mM GDL, which corresponds to a pH of 5.9.

**Figure 3:**
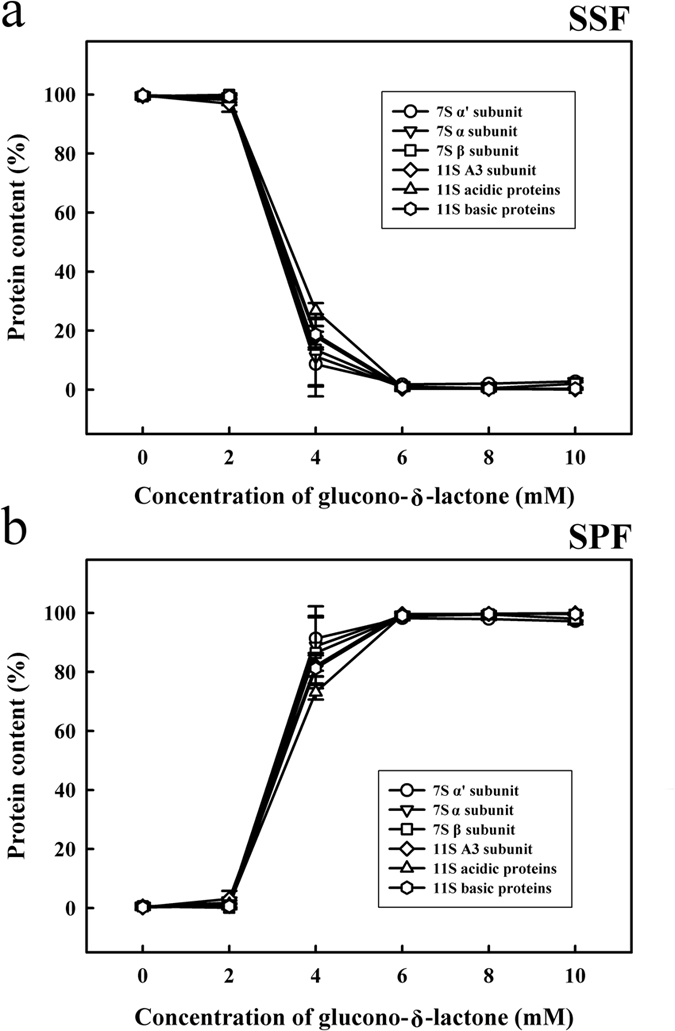
Relative abundances of soymilk proteins treated with various concentrations of GDL (0, 2, 4, 6, 8, or 10 mM) at 85 °C for 45 min: (**a**) Soymilk supernatant fraction (SSF); (**b**) Soymilk pellet fraction (SPF). The results are expressed as protein content (%), which was determined using the Gel-Pro Analyzer software package. Each value is represented as the mean ± standard deviation from measurements conducted in triplicate.

The densitograms corresponding to SDS-PAGE analysis of soymilk samples treated with various quantities of GDL are shown in Fig. 3. As shown in Fig. 3a, the addition of 4 mM GDL decreased the intensities of the basic SSF subunits, as follows: 7S α′ (8.6%), 7S α (11.2%), 7S β (13.5%), 11S A3 (17.9%), 11S acidic (26.8%) and 11S (18.7%). Furthermore, a marked increase in the intensities of the 7S α′, 7S α, 7Sβ, 11S A3 and 11S acids as well as in the 11S basic subunits was observed in the SPF after the addition of 4 mM GDL (Fig. 3b). Our results suggest that the addition of 4 mM GDL could cause the SSF soy proteins, including the 7S α′, 7S α, 7S β, 11S A3 proteins, the 11S acidic protein and the 11S basic protein subunits, to aggregate and form the SPF. Campbell *et al*.^21^ reported that GDL reduces the pH of proteins to a level approaching the isoelectric point of soymilk proteins, which in turn reduces the electrostatic repulsive force and results in the aggregation of low-solubility proteins.

### HPLC analysis of the effects of GDL on the isoflavones in soymilk

**Figure 4:**
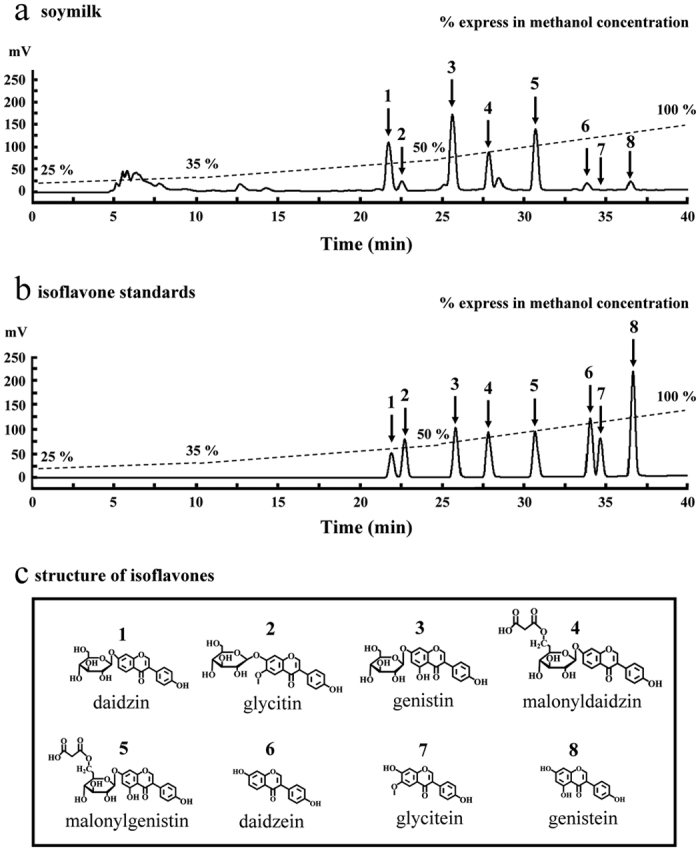
HPLC chromatograms and chemical structures of isoflavones. (**a**) HPLC chromatograms of isoflavones extracted from the soymilk. (**b**) HPLC chromatograms of eight isoflavone standards. Mobile phase: a gradient elution with H_2_O/methanol (75:25, v/v → 0:100, v/v). Flow rate: 0.5 mL/min. Detection: UV 260 nm. Arrow: isoflavones. (**c**) Chemical structures of isoflavones.

As previously mentioned, a total of twelve isoflavones can be found in soymilk. However, the levels of four isoflavones, including malonylglycitin, acetyldaidzin, acetylgenistin and acetylglycitin, are extremely low in soy products. Murphy *et al*.^22^ reported that the quantities in tofu are 0.116, 0.020, 0.051 and 0.041 μmol/g for malonylglycitin, acetyldaidzin, acetylgenistin and acetylglycitin, respectively. Therefore, content of the other eight isoflavones in soymilk were determined by HPLC (Fig. 4). As shown in Fig. 4a, eight isoflavones (arrows, compounds 1–8), including daidzin, glycitin, genistin, malonyldaidzin, malonylgenistin, daidzein, glycitein, and genistein, were isolated and collected from soymilk at elution times of 21–22, 22–23, 25–26, 27–28, 31–32, 33–34, 34–35 and 36–37 min, respectively. The purified daidzin, glycitin, genistin, daidzein, glycitein and genistein were identified by direct comparison with commercial daidzin, glycitin, genistin, daidzein, glycitein and genistein (LC Laboratories, Woburn, MA, USA), while the purified malonyldaidzin and malonylgenistin were identified by direct comparison with commercial malonyldaidzin and malonylgenistin (Nacalai Tesque, Kyoto, Japan). The eight isoflavone standards were shown on the HPLC profile (Fig. 4b). The chemical structure of eight isoflavones is also shown in Fig. 4c. The following sections will focus on the spectral data of the identified eight isoflavones (compounds 1–8) obtained from ESI-MS and UV. Compound 1 (daidzin): ESI-MS: m/z 415.05. UV λ_max_nm: 260; Compound 2 (glycitin): ESI-MS: m/z 468.97. UV λ_max_nm: 262; Compound 3 (genistin): ESI-MS: m/z 468.97. UV λ_max_nm: 262; Compound 4 (malonyldaidzin): UV λ_max_nm: 258; Compound 5 (malonylgenistin): UV λ_max_nm: 260; Compound 6 (daidzein): UV λ_max_nm: 248; Compound 7 (glycitein): UV λ_max_nm: 260; Compound 8 (genistein): UV λ_max_nm: 260. The quantities of specific isoflavones extracted from the soymilk were as follows: daidzin (35.8 ± 0.7 μg/mL), glycitin (4.5 ± 0.1 μg/mL), genistin (30.2 ± 0.7 μg/mL), malonyldaidzin (26.4 ± 0.6 μg/mL), malonylgenistin (44.0 ± 1.0 μg/mL), daidzein (2.2 ± 0.1 μg/mL), glycitein (0.3 ± 0.0 μg/mL) and genistein (1.3 ± 0.1 μg/mL). Wang and Murphy^23^ previously reported that malonyldaidzin and malonylgenistin predominate in soymilk. Jung *et al*.^24^ reported the isoflavone contents extracted from soymilk to be: daidzin (0.50 μmol/g), glycitin (0.14 μmol/g), genistin (0.59 μmol/g), malonyldaidzin (1.55 μmol/g), malonylgenistin (1.34 μmol/g), daidzein (0.31 μmol/g), glycitein (0.07 μmol/g) and genistein (0.26 μmol/g) dry basis. Therefore, daidzin, genistin, malonyldaidzin and malonylgenistin were the major isoflavones in soymilk.

**Figure 5:**
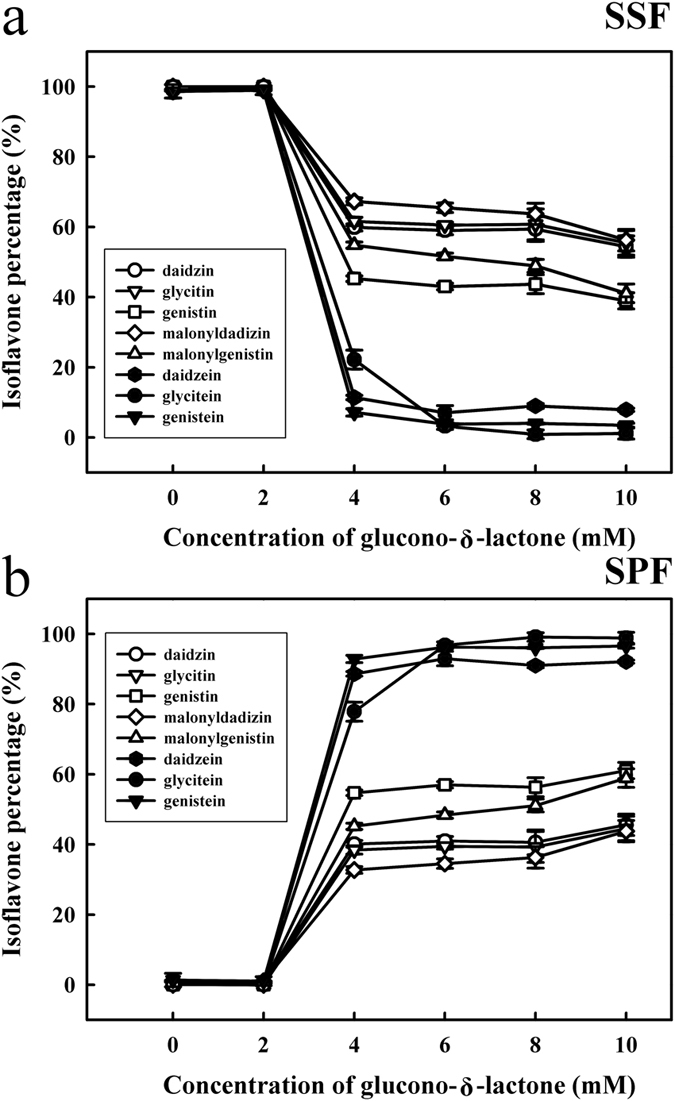
Changes in the isoflavone contents of soymilk with different amounts of GDL (0, 2, 4, 6, 8 or 10 mM) at 85 °C for 45 min. (**a**) soymilk supernatant fraction (SSF); (**b**) soymilk pellet fraction (SPF). Vertical bars represent standard deviations.

The aggregation of isoflavones, including daidzin, glycitin, genistin, malonyldaidzin, malonylgenistin, daidzein, glycitein and genistein, induced by GDL was also evaluated. As shown in Fig. 5, HPLC was used to determine the isoflavone content in SSF and SPF samples with various concentrations of GDL (0, 2, 4, 6, 8 or 10 mM). Figure 5a shows that most of the aglycones, including daidzein, glycitein, and genistein, and a portion of glucosides, including daidzin, glycitin, genistin, malonyldaidzin and malonylgenistin, in the SSF decreased following the addition of 4 mM of GDL. The remaining quantities were as follows: daidzein (11.4 ± 0.6%), glycitein (22.1 ± 2.7%), genistein (7.1 ± 1.0%), daidzin (59.8 ± 0.9%), glycitin (61.5 ± 1.1%), genistin (45.2 ± 0.7%), malonyldaidzin (67.2 ± 0.9%) and malonylgenistin (54.8 ± 0.8%). According to our results, most of the aglycones, including daidzein, glycitein, and genistein, and a portion of glucosides, including daidzin, glycitin, genistin, malonyldaidzin and malonylgenistin, were aggregated from the SSF into the SPF following the addition of 4 mM of GDL (Fig. 5b). However, we noticed that a portion of glucosides, including daidzin, glycitin, genistin, malonyldaidzin and malonylgenistin, were not aggregated from the SSF into SPF. Kao *et al*.^25^ reported that the content of isoflavones in soybean curd (SPF) is influenced by hydrophilic and hydrophobic interactions between soymilk proteins and isoflavones. Aldin *et al*.^26^ reported that the functional group of glucosides make these conjugates more hydrophilic than aglycone isoflavones, which means that they are more likely to be present in the water phase (SSF) than in the protein phase (SPF). The release of isoflavones into the whey (SSF) when soymilk is coagulated to form tofu is associated with the affinity of proteins for isoflavones^27^. As we know, the whey is removed to prepare firm tofu in the tofu-making process. Therefore, our results suggested that a portion of glucosides, including daidzin, glycitin, genistin, malonyldaidzin and malonylgenistin, were not aggregated into tofu (SPF). This is in agreement with Wang and Murphy^23^ who reported losses in the quantity of isoflavones during the processing of soy products.

### HPLC analysis of isoflavones from 7S-isoflavone and 11S-isoflavone complexes

**Figure 6:**
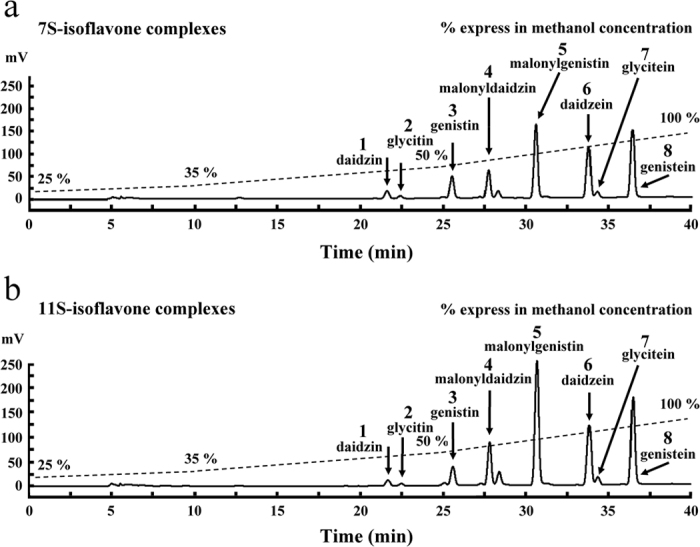
HPLC chromatograms of isoflavones extracted from the (**a**) 7S-isoflavone complexes and (**b**) 11S-isoflavone complexes. Mobile phase: a gradient elution with H_2_O/methanol (75:25, v/v → 0:100, v/v). Flow rate: 0.5 mL/min. Detection: UV 260 nm.

As mentioned above, the proteins in soy include 7S and 11S, which account for approximately 70% of the total protein content. The 7S and 11S bound to isoflavones to form the 7S-isoflavone complexes and 11S-isoflavone complexes in soymilk^16^. Rickert *et al*.^28^ reported that the amounts of daidzin, daidzein and genistein in the 7S proteins were 0.25, 0.43 and 0.54 μmol/g dry basis, respectively. The contents of daidzin, daidzein and genistein in the 11S proteins were 0.62, 0.39 and 0.41 μmol/g dry basis, respectively. The isoflavones extracted from the 7S-isoflavone complexes and the 11S-isoflavone complexes were analyzed by HPLC (Fig. 6). As shown in Fig. 6a, we found that the quantities of isoflavones in 7S proteins were as follows: daidzin (4.76 ± 0.08 μg/mg), glycitin, (0.77 ± 0.02 μg/mg), genistin (6.21 ± 0.09 μg/mg), malonyldaidzin (15.55 ± 0.11 μg/mg), malonylgenistin (40.86 ± 0.34 μg/mg), daidzein (11.47 ± 0.06 μg/mg), glycitein (2.17 ± 0.02 μg/mg) and genistein (7.20 ± 0.03 μg/mg). The fact that these isoflavones could be isolated from 7S proteins indicates that they bonded to 7S proteins to form 7S-isoflavone complexes in soymilk. The isoflavones could be isolated from 11S proteins, and similar results were also observed in the 11S-isoflavone complexes (Fig. 6b). The quantities of isoflavones in 11S proteins were as follows: daidzin (3.16 ± 0.15 μg/mg), glycitin (0.61 ± 0.03 μg/mg), genistin (4.51 ± 0.30 μg/mg), malonyldaidzin (20.75 ± 0.62 μg/mg), malonylgenistin (61.24 ± 1.88 μg/mg), daidzein (11.70 ± 0.33 μg/mg), glycitein (2.62 ± 0.07 μg/mg) and genistein (8.30 ± 0.21 μg/mg). These results are evidence that isoflavones bind to 11S proteins to form 11S-isoflavone complexes in soymilk.

The 7S and 11S proteins were denatured via heating and the hydrophobic areas were exposed during the production of tofu, such that the increased affinity of isoflavones to proteins caused the isoflavones to precipitate in the tofu. Speroni Aguirre *et al*.^29^ reported that the difference in the content of isoflavones between 7S and 11S might be due to structural differences in the proteins, causing a different affinity between proteins and isoflavones. Furthermore, the stronger polarity of glucosides, including daidzin, glycitin, genistin, malonyldaidzin, malonylgenistin, led to their being partitioned into the aqueous phase (SSF), while the weaker polarity of aglycones, including daidzein, glycitein and genistein, remained in SPF^30^. As a result, the co-aggregation of isoflavones with 7S and 11S proteins differed according to the hydrophobic interactions between soymilk proteins and isoflavones as well as with the polarity of the individual isoflavones. Our results suggested that most aglycones, including daidzein, glycitein, and genistein, and a portion of glucosides, including daidzin, glycitin, genistin, malonyldaidzin and malonylgenistin, bound to 7S and 11S to form 7S-isoflavone and 11S-isoflavone complexes in soymilk. These complexes were then aggregated from the SSF into the SPF following the addition of 4 mM of GDL.

### Reaction scheme for the effects of GDL on 7S, 11S and isoflavones

**Figure 7:**
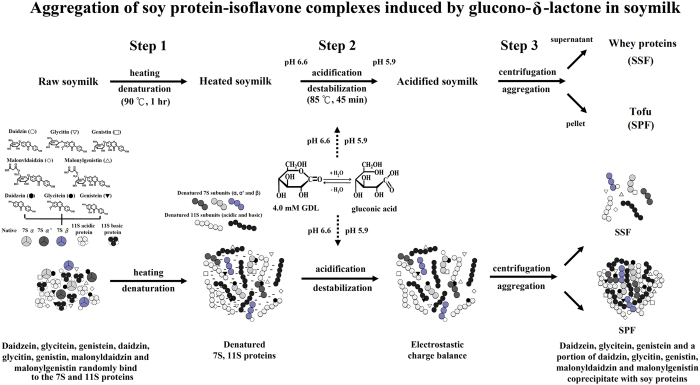
Reaction scheme for the effects of GDL on the aggregation of β-conglycinin, glycinin and isoflavones in soymilk.

Hsiao *et al*.^31^ reported that the heat applied during the processing of soymilk causes the dissociation and denaturation of soymilk proteins, such as 7S and 11S. This process causes the 7S and 11S proteins to unfold and form protein filaments due to the formation of disulfide bonds and hydrophobic interactions. As a result, the surface hydrophobicity of a heated soymilk is higher than that of non-heated soymilk. Furthermore, isoflavones including daidzin, glycitin, genistin, malonyldaidzin, malonylgenistin, daidzein, glycitein and genistein exist freely in soymilk^32^. These isoflavones bind with 7S and 11S proteins to form 7S-isoflavone and 11S-isoflavone complexes in soymilk^16^. Moreover, the addition of GDL to soymilk results in a reduction in soymilk pH. The soymilk protein particles neutralized by acidification reach the isoelectric point. The hydrophobic interactions of the neutralized protein molecules subsequently become predominant and induce aggregation^33^. As the pH of soymilk is decreased to approximately pH 5.8, it indirectly causes soymilk proteins to aggregate via the direct acidification of the soymilk^18^. Based on our results, a potential mechanism for the effect of GDL on the aggregation of soy protein-isoflavone complexes is shown in Fig. 7. It has been suggested that the GDL-induced aggregation reaction includes a three-step process. Heating of the soymilk causes the 7S and 11S proteins to unfold and form protein filaments, which causes the hydrophilic and negatively charged nature of the surface to be lost in the first step. The daidzein, glycitein, and genistein and a portion of daidzin, glycitin, genistin, malonyldaidzin and malonylgenistin were bound with 7S and 11S proteins to form 7S-isoflavone and 11S-isoflavone complexes in the heated soymilk. The second step of the reaction scheme is a process in which 4 mM GDL catalyzes the acidification of the soymilk. The hydrolysis of GDL to gluconic acid results in a decrease in the soymilk pH (pH 5.9). This reduces the electrostatic repulsion between the charged protein filaments and causes them to aggregate. The third step involves the GDL-induced aggregation of 7S-isoflavone and 11S-isoflavone complexes into the SPF. The 7S-isoflavone and 11S-isoflavone complexes yield insoluble complexes, and these complexes contain 7S (α’, α and β subunits), 11S (acidic and basic proteins) and isoflavones, including daidzein, glycitein, genistein, daidzin, glycitin, genistin, malonyldaidzin and malonylgenistin.

In conclusion, we examined the effects of GDL on the aggregation of the 7S, 11S proteins and isoflavones in soymilk. SDS-PAGE analyses showed that 4 mM GDL induced the aggregation of the 7S and 11S proteins. HPLC analysis suggested that most of the daidzein, glycitein, and genistein and a portion of the daidzin, glycitin, genistin, malonyldaidzin and malonylgenistin bound to 7S and 11S proteins to form 7S-isoflavone and 11S-isoflavone complexes. These complexes were precipitated into the SPF by 4 mM GDL, forming soybean curd. The results presented in this study clearly demonstrate that the addition of GDL to soymilk induces the aggregation of 7S, 11S, daidzein, glycitein, genistein, daidzin, glycitin, genistin, malonyldaidzin and malonylgenistin, forming soybean curd. Analytical ultracentrifugation is an appropriate and highly effective method for determining masses of complexes such as those observed in the gel matrix components under study here. Thus, we will be applying these methods to investigate the components of 7S-isoflavone and 11S-isoflavone complexes in a future study.

## Materials and Methods

### Preparation of soymilk

Soybeans [*Glycine max* (L.) Merrill, 100 g] were washed and soaked in distilled water at 25 °C for 12 h. Hydrated seeds were drained and ground into homogenates in 1 L of distilled water. The raw soymilk was filtered through a cotton filter and heated in a 90 °C water bath for 1 h. The soymilk was collected and stored at 4 °C. To investigate the effects of GDL on the aggregation of soymilk proteins, soymilk was centrifuged at 12,000 × g for 10 min at 4 °C using a centrifugal separator to remove lipids. The defatted filtrate was used as soymilk in subsequent experiments.

### Preparation of soymilk samples containing various concentrations of GDL

GDL purchased from Sigma Chemical Co. (St. Louis, MO, USA) was added to soymilk to yield final concentrations of 0, 2, 4, 6, 8 or 10 mM. The mixtures were incubated at 85 °C for 45 min^34^. Following incubation, soymilk samples of 1 mL were fractionated into the soymilk supernatant fraction (SSF) and the soymilk pellet fraction (SPF) by centrifugation for 20 min (12,000× g). SSF samples (1 mL) were collected and SPF samples were resuspended in 1 mL of lysis solution containing 7 M urea, 2 M thiourea, and 4% 3-[(3-cholamidopropyl)-dimethylammonio]-1-propanesulfonate prior to use.

### Determinations of protein concentrations and pH values

The protein concentrations of the soymilk samples were determined using a protein assay kit (Bio-Rad, Hercules, CA, USA). The Bio-Rad protein-assay dye was diluted with 4 volumes of water and then mixed with individual standards or soymilk samples. The absorbance at 595 nm was measured using a VersaMax™ microplate reader (Molecular Devices Corporation, Sunnyvale, CA, USA), and bovine serum albumin (Sigma Chemical Co., St. Louis, MO, USA) was analyzed as the standard. The pH value was measured with a pH meter (Sartorius Basic Meter PB-10, Germany).

### Sodium dodecyl sulfate polyacrylamide gel electrophoresis (SDS-PAGE)

Electrophoresis was performed in 1.5 mm-thick gels with 5% stacking gel (w/v) and 12.5% separating gel (w/v). For each soymilk sample, a 0.1-mL volume of sample was mixed with 0.3 mL of buffer (2% SDS, 5% β-mercaptoethanol, 10% glycerol, 0.02% bromophenol blue and 70 mM Tris-HCl, pH 6.8) and heated to 95 °C for 5 min. The samples (4 μL) and a protein ladder were loaded into separate wells. Following electrophoresis, the gels were stained with Coomassie Brilliant Blue R-250. The stained gels were digitized using an EPSON perfection 1270 image scanner (Epson America Inc., Long Beach, CA, USA) and analyzed using the Gel-Pro Analyzer (version 4.0, Media Cybernetics, Inc.) software program. The level of protein aggregation induced by GDL was evaluated by the magnitudes of the changes in the electrophoretic profiles.

### Preparation of 7S-isoflavone and 11S-isoflavone complexes

The 7S-isoflavone and 11S-isoflavone complexes were isolated from soymilk using a method modified from Hsiao *et al*.^16^ as follows. Briefly, 0.1% sodium bisulfate was added to soymilk (25 mL) and the pH was adjusted to 6.4 with 0.2 M HCl and then incubated at 4 °C for 12 h. After 20 min centrifugation at 6,500× g at 4 °C, the pellet (11S protein-isoflavone complexes) was collected. NaCl (0.25 M) was added to the supernatant, and the pH was adjusted to 5.0 with 0.2 M HCl and incubated at 4 °C for 1 h. After 30 min centrifugation at 9,000× g at 4 °C, the supernatant was collected and adjusted to pH 4.8 with 0.2 M HCl. After 20 min centrifugation at 6,500× g at 4 °C, the pellet (7S protein-isoflavone complexes) was collected.

### Preparation of isoflavone samples

Soymilk, SSF, SPF, 7S-isoflavone complexes and 11S-isoflavone complexes samples were lyophilized into powder to extract isoflavones in accordance with the method presented by Prabhakaran *et al*.^35^. Eighty percent methanol was added to freeze-dried soymilk samples to serve as an extraction solvent, and the solution was shaken in a vortex mixer at 30 °C for 2 h. After 10 min centrifugation at 12,000× g at 4 °C, the supernatants (isoflavones) were subjected to HPLC analysis.

### HPLC analysis and identification of the isoflavones

Analysis of isoflavone samples (20 μL) was performed using a method modified from Tipkanon *et al*.^36^. The HPLC system comprised a PU-980, JASCO pump, UV-970, JASCO detector, and a C18 packed column (Mightysil RP-18 GP, 4.6 mm × 250 mm, 5 μm Spherical, Kanto Chemicals, Tokyo, Japan). The mobile phase of methanol (A) and water (B) with a gradient elution of 25% A and 75% B increased to 35% A in 10 min, 50% A in 25 min, 100% A in 35 min, and 100% A in 40 min. The flow rate was 0.5 mL/min with the column maintained at a temperature of 30 °C. Detection was conducted at a wavelength of 260 nm. The isoflavones were collected and their chemical structures were identified using ultraviolet spectra (UV, Hitachi, U-1900) and high-resolution ESI-TOF mass spectrometry (ESI-MS, BioTOF III; Bruker Daltonics, Inc. Billerica, MA, USA).

### Statistical analysis

Data were expressed as the means ± standard deviations. The data were analyzed using Statistical Package for the Social Sciences software (SPSS for Windows, version 10.0.7C, SPSS Inc., Chicago, IL, USA). The statistically significant differences between treatments were determined using one-way ANOVA followed by Duncan’s multiple range test. Three determinations for each treatment were performed, and the significance level was set at *P* < 0.05.

## Additional Information

**How to cite this article**: Hsia, S.-Y. *et al*. Aggregation of soy protein-isoflavone complexes and gel formation induced by glucono-δ-lactone in soymilk. *Sci. Rep.* **6**, 35718; doi: 10.1038/srep35718 (2016).
